# Pt-Free Counter Electrodes with Carbon Black and 3D Network Epoxy Polymer Composites

**DOI:** 10.1038/srep22987

**Published:** 2016-03-10

**Authors:** Gyeongho Kang, Jongmin Choi, Taiho Park

**Affiliations:** 1 Department of Chemical Engineering, Pohang University of Science and Technology, Pohang 790-780, Republic of Korea

## Abstract

Carbon black (CB) and a 3D network epoxy polymer composite, representing dual functions for conductive corrosion protective layer (CCPL) and catalytic layer (CL) by the control of CB weight ratio against polymer is developed. Our strategy provides a proper approach which applies high catalytic ability and chemical stability of CB in corrosive triiodide/iodide (I_3_^−^/I^−^) redox electrolyte system. The CB and a 3D network epoxy polymer composite coated on the stainless steel (SS) electrode to alternate counter electrodes in dye sensitized solar cells (DSSCs). A two-step spray pyrolysis process is used to apply a solution containing epoxy monomers and a polyfunctional amine hardener with 6 wt% CB to a SS substrate, which forms a CCPL. Subsequently, an 86 wt% CB is applied to form a CL. The excellent catalytic properties and corrosion protective properties of the CB and 3D network epoxy polymer composites produce efficient counter electrodes that can replace fluorine-doped tin oxide (FTO) with CCPL/SS and Pt/FTO with CL/CCPL/SS in DSSCs. This approach provides a promising approach to the development of efficient, stable, and cheap solar cells, paving the way for large-scale commercialization.

For more than two decades, dye-sensitized solar cells (DSSCs) have been intensively investigated in industry and academia as a viable alternative to conventional silicon-based photovoltaic cells^1^,^2^,^3^,^4^,^5^,^6^,^7^,^8^,^9^,^10^. A typical DSSC comprises a dye-sensitized titanium dioxide (TiO_2_) nanocrystalline photoanode, an electrolyte that provided a redox couple (I_3_^−^/I^−^), and a counter electrode (CE)^11^. Significant research efforts have been applied toward optimizing each component by modifying the photoanodes, developing alternative dyes, improving the redox couple, and introducing structural changes, to yield highly efficient DSSCs^12^,^13^,^14^,^15^,^16^,^17^,^18^,^19^. Pt-coated fluorine-doped tin oxide (FTO) is typically used as the CE because of its excellent catalytic activity toward reducing I_3_^−^. Unfortunately, Pt-coated CE is typically produced through high-temperature hydrolysis processes that are incompatible with flexible conducting polymer electrodes^20^,^21^,^22^,^23^,^24^. Furthermore, Pt corrodes in electrolytes containing iodide to generate PtI_4_^24^,^25^,^26^. The Pt metal catalyst coating has been replaced with other materials, such as titanium nitrides 10,27, cobalt sulfide^28^, and carbon derivatives^29^,^30^,^31^,^32^,^33^. Carbon derivatives are particularly attractive, as they are abundant, low-cost, and provide high catalytic activities while remaining chemically stable in the presence of iodine redox couples^32^,^33^,^34^,^35^,^36^. Carbon black (CB) has been used on a large scale, for example in printing toners, reinforcing additives in automobile tires, and as conductive fillers in plastics, elastomers, and films^37^. CB is a conductive material with good catalytic activity for the reduction of triiodide^38^. Carbonaceous materials present active catalytic sites at their edges^39^. Therefore, CB, which have many edges, may be more active than highly structured carbon materials, such as graphites, graphenes, and carbon nanotubes^40^,^41^. CB powders cannot retain their shapes on a substrate; thus, they must be mixed with other binder materials, such as polymers or a TiO_2_ slurry containing organic surfactants and binders^34^,^35^,^42^. For example, Grätzel *et al.*^40^ and Jun *et al.*^43^, independently employed a CB/TiO_2_ slurry system in DSSC electrodes. Recently, Ho *et al.* reported the use of a CB/polymer composite CE comprising polypyrrole, polyaniline, or poly(3,4-ethylenedioxythiophene^44^. We, herein, extend the approaches introduced in these works by examining three-dimensional (3D) network polymers formed by polymerized monomers that had been cross-linked (XL) *in-situ* with CB to robustly fix the CB powders onto a substrate and act as a binder for the CB powders. The 3D network polymers are composed of an epoxy monomer and a polyfunctional amine hardener. The chemically XL epoxy polymer provides strong mechanical properties, chemical resistance, thermal resistance, and adhesive properties^45^,^46^.

Here, we describe the development of novel stainless steel (SS) CEs coated with a composite of CB and 3D networked polymers to replace conventional Pt CEs on FTO glass. Previous studies have reported the development of FTO glass alternates for the fabrication of flexible and bendable electrodes^47^,^48^. SSs are highly conductive and have a work function (–4.4 eV) that is appropriate for use as a CE in DSSCs^49^. SSs are cost-effective materials compared with FTO glasses. Tan *et al.*^50^ and Kang *et al.*^51^ independently reported the development of an SS mesh and an SS substrate-based CE, respectively. However, the previous reports did not comment on the corrosion problems inherent to these materials and not show the long-term stability of the resulting DSSCs. Corrosion is a key technical feature that must be addressed in practical applications of SS CEs in DSSCs. In this work, we report a CB-coated SS CE with highly thermal stability, thus without corrosion problems. The photovoltaic performance for the device using this CB-coated SS CE remained nearly constant after 30 days of storage at 65 °C. Meanwhile, a reference device consisting of Pt/FTO decreased up to almost 50% of the photovoltaic performance. We successfully coated both a conductive corrosion protective layer (CCPL) and a catalytic layer (CL) with a composite of CB and 3D network polymers onto an SS substrate using spray pyrolysis, as illustrated in Fig. 1a. The weights ratio of the CB incorporated into the polymer matrices of the CCPL and CL were 6 and 86wt%, respectively. The CCPL formed a compact dense structure that prevented corrosion due to electrolyte penetration, whereas the CL formed a nanoporous structure that provided a large surface area for increased catalytic activity.

## Results

### Percolation concentration of CB in CCPL

**Figure 1:**
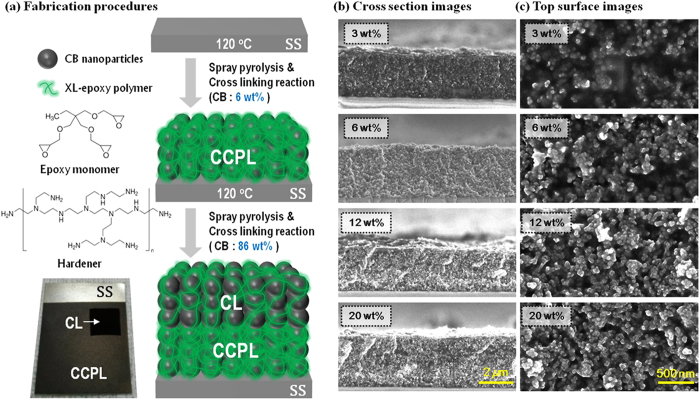
(**a**) Illustration of the procedure used to fabricate the SS counter electrode containing a composite of CB and the 3D network polymers, and the chemical structures of trimethylolpropane triglycidyl ether (epoxy monomer) and polyethylenimine (hardener). A two-step spray pyrolysis process using a low wt% CB, followed by a high wt% CB, was used to prepare a conductive corrosion protective layer (CCPL) and a catalytic layer (CL). The *in-situ* cross-linking network polymerization reaction occurred instantly as soon as the mixed solution was sprayed onto a hot SS substrate or a hot CCPL. Cross-sectional images (**b**) and top-view images (**c**) obtained using field emission scanning electron microscopy (FE-SEM). Shown are the various composites prepared using CB and the 3D network polymers. Ash-color (or white) nanoparticles and the dark matrix represent dispersed CB and the 3D network polymers, respectively.

We optimized a coating composition containing CB and a polymer for use as a CCPL by preparing 4 samples (3, 6, 12, and 20 wt%), and we investigated their morphology, conductivity, corrosion protection properties, and electrical and catalytic properties. Figure 1b,c shows field emission scanning electron microscopy (FE-SEM) images of the CCPL layer cross-section and top surface, respectively. Smooth films with 2.4–2.5 μm thick were obtained, and the CB are well-dispersed within the 3D XL polymer matrix.

**Figure 2:**
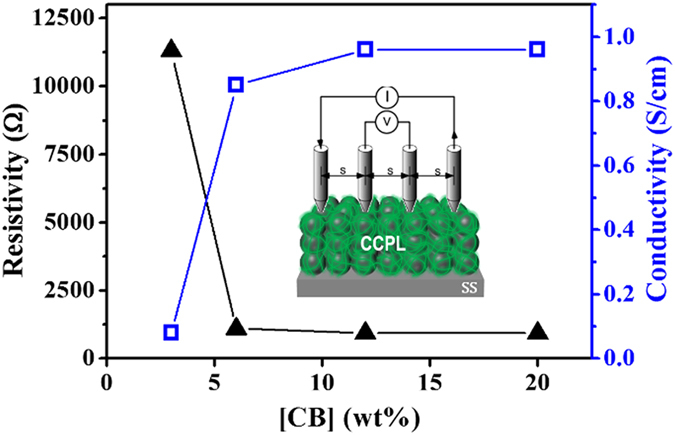
The resistivity and conductivity as a function of the wt% CB.

The composites prepared with more than 6 wt% CB displayed well-established vertical interconnectivity among the CB due to the *in-situ* 3D XL polymer matrix, which appeared to improve as the CB content increase. The CB film did not detach from the composite during the pressure sensitive tape (PST) test. These results indicated that the XL polymers acted as an excellent binder for the CB. An efficient charge transport was obtained in the compact CB composites, and the *in-situ* generated 3D XL polymers guaranteed interconnections among the CB. Samples in which the CB were separated as a result of the large polymer content yield poor charge transport through the conducting CB network. Good mechanical properties, including adhesion, chemical resistance, and corrosion protection, were obtained at optimal composite compositions (e.g., at the percolation concentration) without incurring a loss in the conductivity^52^. The resistivities of the four composites using a 4-point probe method (see the inset in Fig. 2) were measured and the conductivities were calculated (Table 1).

The conductivity of the composite prepared 3 wt% CB was 0.081 S cm^−1^, almost one order of magnitude lower than the conductivity of a film prepared with 6 wt% CB (0.85 S cm^−1^). Further increases in the CB content only increased the conductivity to 0.96 S cm^−1^ at 20 wt% CB. Figure 2 shows the CB concentration-dependent conductivity. A critical point indicative of the percolation concentration was reached at 6 wt% CB. Therefore, we employed 6 wt% CB in the CCPL tested in this work.

### Chemical resistance and corrosion protective properties of the CCPL

**Figure 3:**
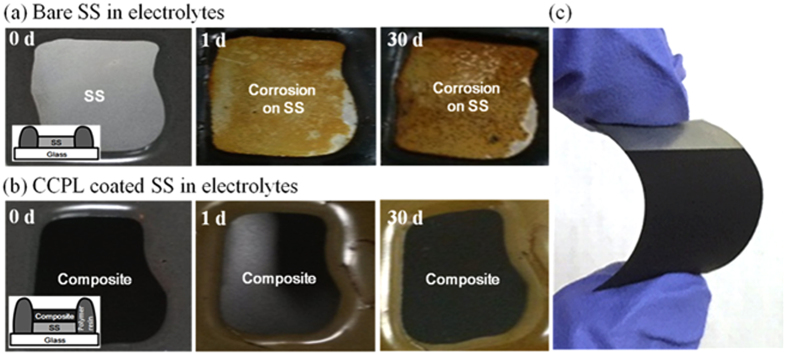
Comparison of the corrosion level of (**a**) the bare SS and (**b**) the CCPL coated SS against an electrolyte solution containing iodine redox couples. Insets: schematic diagram showing the structures of the samples. (**c**) A bent image of a conductive corrosion protective layer (CCPL) coated stainless steel (SS) electrode.

We next investigated the chemical resistance and corrosion protective properties of the CCPL (6 wt%) in the presence of an electrolyte solution and compared these properties to those obtained using a bare SS substrate dipped in an electrolyte containing 0.03 M iodine and additives (t-butyl pyridine (tBP) and lithium bis(trifluoromethanesulfonyl)imide (LiTFSI)) at 65 °C. The bare SS substrate was completely corroded within a day, as indicated by the observed color change (Fig. 3a). On the other hand, no corrosion was observed at the CCPL surface, even after 30 days (Fig. 3b). We attempted to compare the bare SS surfaces before and after the soaking test by removing the CCPL layer, but the adhesion between the SS and CCPL layer was too robust to permit removal of the CCPL. These results demonstrated that the CCPL do not react with the electrolyte, and the CCPL effectively prevented the liquid electrolyte from reaching the SS. A 6 wt% CB solution provided the percolation concentration in our system which provided good adhesive properties, good chemical resistance and good corrosion protection ability without a loss in conductivity. In addition, the CCPL/SS electrode exhibited high flexibility due to the high ductility of a composite of CB and 3D network polymers on an SS substrate. Figure 3c shows no cracks under bending deformation of more than 100 times.

### Redox activity of CCPL

**Figure 4:**
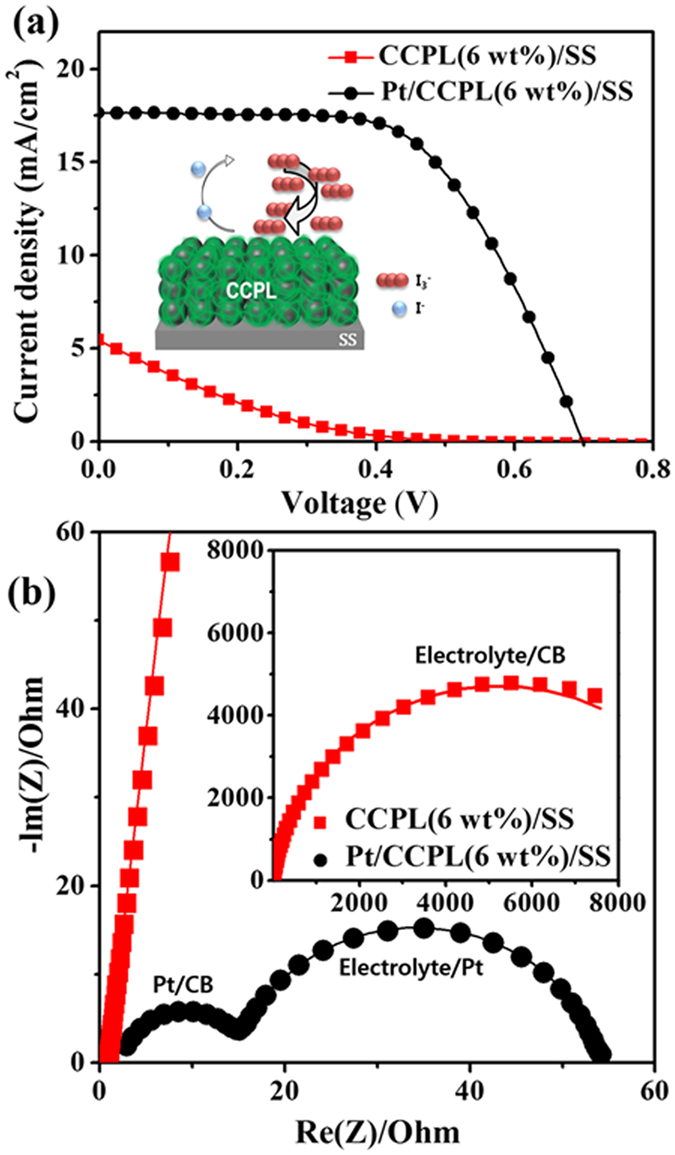
(**a**) Representative *J*–*V* curves indicating the photovoltaic performances of DSSC prepared with CCPL/SS and Pt/CCPL/SS CEs under AM 1.5 illumination. (**b**) Electrochemical impedance spectra of the CCPL/SS and Pt/CCPL/SS CEs in symmetric cells consisting of two identical CEs at 0 V under dark conditions.

Devices having a compact CCPL layer were firstly fabricated to evaluate the catalytic properties of the CCPL because the electrode (CCPL/SS) prepared with 6 wt% CB exhibited a reasonably high conductivity. In addition, we prepared a control CE based on a Pt-coated CCPL layer (Pt/CCPL/SS) for comparison with the CCPL/SS electrode. Figure 4a shows the *J*–*V* characteristics of the devices prepared with the CCPL/SS and Pt/CCPL/SS CEs. The devices employing the CCPL CE exhibited a very poor photovoltaic performance with a short-circuit current density (*J*_*SC*_) of 5.5 mA cm^−2^, an open-circuit voltage (*V*_*OC*_) of 0.53 V, a fill factor (FF) of 14.9%, and a power conversion efficiency (PCE) of 0.43%. These values were lower than those obtained from the device employing the Pt/CCPL/SS CE (*J*_*SC*_ = 17.7 mA cm^−2^, *V*_*OC*_ = 0.70 V, FF = 59.4%, and PCE = 7.3%). These results indicated that the compact CCPL CE could not function as both a corrosion protective layer and as a catalytic layer due to the insufficient contact area between the CB nanoparticles and I_3_^−^. A good photovoltaic performance was obtained from the device prepared with the Pt/CCPL/SS CE, demonstrating that FTO can be replaced with a CCPL/SS substrate. These results suggested that the electron transport from the Pt layer to the SS through the interconnected CB in the CCPL was highly efficient. Figure 4b shows the electrochemical impedance spectroscopy (EIS) results of CCPL/SS and Pt/CCPL/SS CEs in symmetric cells consisting of two identical CEs (see Figure S1 for the equivalent circuit models). The impedance spectrum of the Pt/CCPL/SS CE was dominated by the I_3_^−^/I^−^ redox reaction at Pt and the charges transfer to the SS through the CB in the CCPL. Assuming that the Pt/CB and CB/SS interfaces were characterized as non-Ohmic and Ohmic contacts, respectively, two different interfaces were expected to be present: the electrolyte/Pt and Pt/CB interfaces. Indeed, two semicircles were observed in the spectra. The small semicircle at high frequencies and the large semicircle at low frequencies (in the dark, under a forward bias) were attributed to electron transfer (series resistance, R_S_ = ca. 11 Ωcm^2^) at the Pt/CB interface and the redox reaction (charge transfer resistance, R_CT_ = ca. 40 Ωcm^2^) at the electrolyte/Pt interface, respectively. These results indicated that the catalytic activity of Pt is good, although electrons were more easily transport from Pt to the CB than from Pt to the electrolyte during catalysis. A symmetric cell consisting of two CCPL/SS CEs exhibited only one semicircle characterized by 10,500 Ωcm^2^ at the electrolyte/CB interface due to the Ohmic contact at the CB/SS interface; however, the R_CT_ value is large, indicating poor catalytic properties at the CCPL CE. This result agree well with the poor photovoltaic performances of the devices prepared with only the CCPL CE, suggesting that a catalytic layer (CL) with a nanoporous structure was required to increase the surface area at the electrolyte/CB interface.

### Adhesive property of the CL

The CB content was increased (and the polymer content decreased) to increase the surface area of the CB. This could decrease the adhesion properties of the CB on the 3D XL polymer matrix. The maximum CB content at which the CB did not detach from the surface of the CL was identified by conducting a pull-off adhesion strength test using a PST to evaluate the adhesive properties of the CLs having CB contents up to 95 wt%. Figure S2 shows representative photographs of the pull-off adhesion strength test results obtained from the two CLs containing 86 or 90 wt% CB. The detachment of CB on the surfaces of the PST was not observed until an 86 wt% CB is used in the CLs. A greater number of CB were found to detach at concentrations beyond this threshold. The detached CB were clearly visible on the PST at and above a 90 wt% CB in the CL, as indicated by the yellow dotted circle in the photograph of the PST surface. These results were used to determine a CB concentration of 86 wt% in the optimized CL on the CCPL.

### Electrochemical catalytic ability of the CL

**Figure 5:**
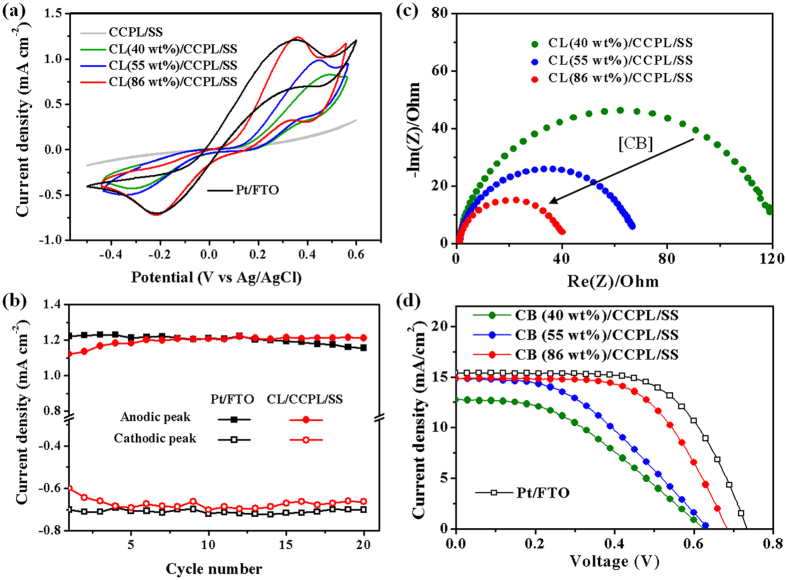
(**a**) Cyclic voltammograms obtained from the oxidation and reduction of the I_3_^−^/I^−^ redox couple using the CCPL/SS, CL(40, 55 or 86 wt%)/CCPL/SS or Pt/FTO electrodes, at a scan rate of 30 mV/s. (**b**) Anodic and cathodic peak current densities for 20 consecutive CVs of the Pt/FTO electrode and the CL(86wt%)/CCPL/SS electrode. (**c**) Electrochemical impedance spectra of the CL/CCPL/SS ECs prepared with 40%, 55%, or 86% CB in symmetric cells consisting of two identical CEs at 0 V under dark conditions. (**d**) Representative J–V curves measured in devices prepared using CL/CCPL/SS ECs with 40%, 55%, or 86% CB, and a Pt/FTO CE as a control.

In order to investigate the electrochemical catalytic abilities of the CL, cyclic voltammetry (CV) measurement was performed using a three-electrode electrochemical system with a scan rate of 30 mV/s. The working electrodes were CL/CCPL/SS electrodes with 40, 55 or 86 wt% CB in the CLs. Pt/FTO and CCPL/SS electrodes also tested for comparison. A Pt wire and Ag/AgCl were used as a counter electrode and a reference electrode, respectively, in the acetonitrile solution. As shown in Fig. 5a, each CV curve presents one pair of redox peaks corresponded to the reaction of I_3_^−^ + 2e^−^ ↔ 3I^−^. An anodic peak current density (*J*_pa_) and a cathodic peak current density (*J*_pc_) corresponded to the oxidation of the iodide ions and the reduction of the tri-iodide ions, respectively. The oxidation/reduction peaks for the Pt/FTO electrode were shown at 0.352 V/−0.210 V with peak to peak separation potential (*E*_pp_) of 0.562 V and *J*_pa_/*J*_pc_ values were 1.211 mA cm^−2^/−0.700 mA cm^−2^. The narrower *E*_pp_ and higher *J*_p_ indicated better electrochemical catalytic ability. In the CCPL/SS electrode, very small amounts of currents flew through the surface of CCPL which had a compact dense structure with a small surface area. The small amounts of currents account for a high R_CT_ value obtained from EIS measurement. CV results of electrodes with increased CB weight ratio (40, 55 or 86 wt%) in the CL presented decreased *E*_pp_ and enlarged *J*_p_ values, indicating effective electrochemical catalytic abilities. The increase of weight ratio of CB in the CL resulted in a higher surface area, where redox reaction occurred, inducing the smaller R_CT_. The *E*_pp_, *J*_pa_ and *J*_pc_ values of the CL(86wt%)/CCPL/SS electrode were 0.576 V, 1.206 mA cm^−2^ and −0.701 mA cm^−2^, respectively. This alternative electrode exhibited the efficient charge transfer ability, which was comparable with the Pt/FTO electrode by the decline of R_ct_. Figure 5b shows the *J*_pa_ and *J*_pc_ of the Pt/FTO electrode and the CL(86wt%)/CCPL/SS electrode during the 20 consecutive cycle scans. The *J*_p_ values of the CL/CCPL/SS electrode were almost maintained steadily, which revealed the excellent electrochemical stability in the I_3_^−^/I^−^ based electrolyte system.

### Photovoltaic performances and impedance analysis of DSSCs

As demonstrated above, the electrochemical impedance spectroscopy (EIS) measurements obtained from the symmetric cells established the criteria for the CL surface activity. Figure 5c shows the EIS spectra obtained from symmetric cells consisting of CL/CCPL/SS CEs with 40, 55 or 86 wt% CB in the CLs. One semicircle corresponded to Ohmic contact between the CB in the CL and the CCPL. The resistance of the CL (40 wt%) due to the catalytic activity at the electrolyte/CB interface was R_CT_ = 119 Ω cm^2^, much smaller than the resistance obtained from the CCPL (10,500 Ω cm^2^ for 6 wt% CB). This value decreased as the CB concentration increase. R_CT_ for the CB (86 wt%) is 41 Ω cm^2^, almost equal to the value obtained from Pt/CCPL (40 Ω cm^2^, Table 1).

Photocells employing CL/CCPL/SS CEs having 40, 55, or 86 wt% CB in the CLs were fabricated, and the *J*-*V* characteristics under simulated air mass 1.5 global (AM 1.5G) solar irradiation were measured. The photovoltaic parameters, measured resistance values and catalytic activities are summarized in Table 2, and the representative *J*–*V* characteristics are presented in Fig. 5d. The photovoltaic performances of the devices followed the trends in the R_CT_ values, indicating that the catalytic activity at the electrolyte/CB interface could be enhanced by increasing the CB content in the CL of the CL/CCPL/SS CE. As the CB content in the CL increased, the FF values increased from 40.3 to 60.6% due to the enhanced catalytic activity, which minimized the recombination reaction between photoinduced electrons and I_3_^−^. The improved catalytic activity affected the *V*_*OC*_ values according to the equation: *V*_*OC*_ ~ (nkT/q) × ln(*J*_*SC*_/*J*_*S*_), where, n is the device ideality factor, k is the Boltzmann constant, T is the temperature in Kelvin, q is the fundamental charge, and *J*_*S*_ is the saturation current density^53^,^54^.

The best performance was obtained from the device prepared with a CL(86 wt%)/CCPL/SS CE, yielding *J*_*SC*_ = 14.9 mA cm^–2^, *V*_*OC*_ = 0.68 V, FF = 60.6%, and PCE = 6.1%. With the exception of *J*_*SC*_, these values were equal to those obtained from a device prepared with a Pt/CCPL/SS CE (*J*_*SC*_ = 17.7 mA cm^−2^, *V*_*OC*_ = 0.70 V, FF = 59.4%, and PCE = 7.3%) as summarized in Table 1. In general, the *J*_*SC*_ values could be estimated using the equation: *J*_*SC*_ = q × η_lh_ × η_inj_ × η_cc_ × I_o_, where q is the fundamental charge of an electron, I_o_ is the intensity of the incident light, η_lh_ is the light harvesting efficiency, η_inj_ is the electron injection efficiency from the excited dye molecules to the TiO_2_ conduction band, and η_cc_ is the charge collection efficiency of the injected electrons at the transparent conductive oxide (TCO) layer. Assuming that I_o_, η_inj_, and η_cc_ were equal in the two devices, different *J*_*SC*_ values could account for the variations in the quantity of dye molecules adsorbed onto the electrodes, as reflected in η_lh_, although we employed identical experimental conditions (Figure S3). The different counter electrodes in this study did not significantly affect the *J*_*SC*_ values once a reasonably high FF had been achieved due to the effective catalytic properties.

We further compared the performance properties of the CL(86 wt%)/CCPL/SS CE with a conventional Pt/FTO CE. The device prepared with a Pt/FTO CE afforded *J*_*SC*_ = 15.4 mA cm^–2^, *V*_*OC*_ = 0.74 V, FF = 62.9%, and PCE = 7.1%). Interestingly, the *V*_*OC*_ value was slightly larger than that (0.68 V) obtained from the CL(86 wt%)/CCPL/SS CE. As mentioned above, the *V*_*OC*_ value might be sensitive to the *J*_*SC*_ drop caused by the recombination reaction due to the relatively ineffective catalytic activity of the CL(86 wt%)/CCPL/SS CE compared to the Pt/FTO CE; however, the FF and *J*_*SC*_ values of the CL(86 wt%)/CCPL/SS CE were similar to those measured in the Pt/FTO CE; thus, the small *V*_*OC*_ value measured in the CL(86 wt%)/CCPL/SS CE could not be attributed to the *J*_*SC*_ drop alone. The theoretical *V*_*OC*_ could be calculated from the energy difference between the quasi-Fermi level of a nanocrystalline TiO_2_ working electrode under illumination and the redox potential of the I_3_^−^/I^−^ pair in the electrolyte. The redox potential of the electrolyte (E_redox_) is given by the Nernst equation: E_redox_ = E_0_ + (RT/2F)ln([I_3_^−^]/[I^−^]^3^), where E_0_ is the formal potential, R is the gas constant, T is the absolute temperature, and F is the Faraday constant. In addition to the *J*_*SC*_ drop, the small *V*_*OC*_ value obtained from the CL(86 wt%)/CCPL/SS CE may have accounted for the increased E_redox_ due to changes in the redox species concentration at the surface of the dye-sensitized TiO_2_ photoanode.

We previously observed that I_3_^−^ molecules became entrapped in the highly porous network polymer matrix at a TiO_2_ photoanode, thereby increasing *V*_*OC*_. Here, the opposite conditions were present because we employed a porous 3D network polymer matrix at the CE. These conditions were consistent with the measured decrease in *V*_*OC*_ as the polymer content in the CLs increased, which increased the number of sites available to trap I_3_^-^. Nevertheless, the PCE of the device prepared with the CL(86 wt%)/CCPL/SS electrode reached a PCE that is 86% of the PCE obtained from a device prepared with Pt/FTO. Our results suggest that FTO could be replaced with CCPL/SS, but also that Pt/FTO could be replaced with CL/CCPL/SS.

In conclusion we successfully fabricated a novel flexible and cost-effective stainless steel (SS) counter electrode from a composite composed of CB and 3D network polymers to function as a CCPL and CL. The composite film, which provided a catalytic layer and acted as a conductive corrosion protective film, was prepared using a mixture of CB, cross-linkable epoxy monomers, and polyamine hardener and was applied using the spray pyrolysis method. The SS substrate acted as an efficient charge collecting electrode. The CCPL (6 wt% of CB) was chemically stable against electrolytes and successfully prevent the penetration of electrolytes, demonstrating excellent corrosion protective properties. The Pt/CCPL/SS CE was successfully substituted for a conventional Pt/FTO CE and displayed a photovoltaic performance (PCE = 7.3%) that was equivalent to that obtained from PT/FTO (PCE = 7.1%). The CL (86% of CB) provided an effective substitute for the conventional catalytic material (Pt). The DSSC prepared using the CL/CCPL/SS CE also displayed a high photovoltaic performance (PCE = 6.1%). We demonstrated that the CB-coated SS CE (CL/CCPL/SS CE) exhibited highly thermal stability, thus there were no corrosion problems. Furthermore, these results demonstrated that FTO can be replaced with CCPL/SS and Pt/FTO can be replaced with CL/CCPL/SS. Further performance enhancements may be realized by carefully optimizing the device architecture.

## Methods

### Chemicals and materials

Stainless steel (SS) was provided from POSCO. Carbon black (CB) powders purchased from Beilum Carbon Chemical Limited. Polyethylenimine (branched, average Mw ~25,000 by light scattering method, average Mn ~10,000 by GPC), trimethylolpropane triglycidyl ether, tert-butanol, iodine (I_2_), guanidinium thiocyanate (GuSCN), 4-tert-butylpyridine (tBP), acetonitrile (AN), and valeronitrile (VN) were purchased from Sigma-Aldrich. Fluorine-doped SnO_2_ (FTO) glass, Ti-nanoxide T/SP. Ti-nanoxide R/SP, cis-diisothiocyanato-bis(2,2′-bipyridyl-4,4′-dicarboxylato) ruthenium(II) bis(tetrabutyl-ammonium) (N719), 1-butyl-3-methylimidazolium iodide (BMII) were purchased from Solaronix SA. Spray gun (AIR BRUSH KIT, ABS-130) was purchased from Bluebird.

### Preparation of the stainless steel counter electrode with a composite of CB and cross linked (XL) 3D network polymers

A SS substrate was cleaned using water and ethanol, then dried using air blow. The cleaned SS was placed onto the hotplate at 120 °C. Trimethylolpropane triglycidyl ether and polyethylenimine was dissolved in ethanol. Various wt% (3, 6, 12, 20, 40, 55, 86 and 90 wt% ) of CB was dispersed in the solution through ultrasonication (750 W, Sonics & Materials, Inc, Newtown, CT, USA) for 10 min. Firstly, the mixed solution with small wt% of CB to polymer was sprayed onto the SS substrate to form a CCPL. Subsequently, the mixed solution with large wt% of CB to polymer was sprayed onto the CCPL to form a CL. Each spray pyrolysis step was taken for 5 minutes in speed of 2 mL min^−1^. *In-situ* cross-linking network polymerization instantly occurred as soon as the mixed solution sprayed at the hot SS substrate or the hot CCPL.

## Additional Information

**How to cite this article**: Kang, G. *et al.* Pt-Free Counter Electrodes with Carbon Black and 3D Network Epoxy Polymer Composites. *Sci. Rep.* **6**, 22987; doi: 10.1038/srep22987 (2016).

## Supplementary Material

Supplementary Information

## Figures and Tables

**Table 1:** Thickness (μm), resistivity (Ohm), sheet resistance (Ohm/◻), and conductivity values obtained from the CCPL/SS substrates prepared with 3, 6, 12 or 20 wt% CB.

CB (wt%)	Thickness^a^ (μm)	Resistivity^b^ (ohm)	Sheet resistance^c^ (ohm/◻)	Conductivity (S/cm)
3	2.4	11300	51000	0.081
6	2.4	1100	4900	0.85
12	2.5	930	4200	0.96
20	2.5	930	4200	0.96

^a^Values were obtained from the average heights measured from the cross-sectional FE-SEM images.

^b^Determined using the 4-point probe method. ^c^ Sheet resistance = correction factor × resistivity obtained from the 4-point probe method. The value of the correction factor was 4.532, the conducting film thickness was less than the distance of the spacing between the probe tips (1000 μm), and the edges of the film (2.5 cm) were separated from the measurement point by more than 4 times the distance between the probe tips.

**Table 2:** Summary of the catalytic resistance values obtained from various electrodes and their photocurrent–voltage characteristics^a^ under AM 1.5 illumination.

Electrodes	R_CT_^b^ (Ω cm^2^)	*J*_*SC*_ (mA/cm^2^)	*V*_*OC*_ (V)	FF (%)	PCE (%)
CCPL(6 wt%)/SS	10,500	5.5	0.53	14.9	0.43
Pt/CCPL(6 wt%)/SS	40	17.7	0.70	59.4	7.3
CL(40 wt%)/CCPL/SS	119	12.7	0.62	40.3	3.2
CL(55 wt%)/CCPL/SS	68	14.8	0.64	42.7	4.0
CL(86 wt%)/CCPL/SS	41	14.9	0.68	60.6	6.1
Pt/FTO	—	15.4	0.74	62.9	7.1

^a^10 devices prepared using each CE.

^b^The catalytic resistances (R_CT_) were calculated by fitting to an equivalent circuit (the models are illustrated in Figure S1).
