# Supplementary material for: Pt-Free Counter Electrodes with Carbon Black and 3D Network Epoxy Polymer Composites
Source: Sci Rep. 2016 Mar 10;6:22987. doi: 10.1038/srep22987 (PMC4785350; doi:10.1038/srep22987)
Supplement: Supplementary Information [file srep22987-s1.pdf]

Supporting Information

For

Pt-Free Counter Electrodes with Carbon Black and 3D network epoxy polymer composites

Gyeongho Kang, Jongmin Choi, and Taiho Park*

[*] Prof. Taiho Park. Corresponding-Author

Pohang University of Science and Technology (POSTECH),

77 Cheongam-Ro, Nam-gu, Pohang, Kyoungbuk, Korea. Fax: +82-54-279-8298;

Tel: +82-54-279-2394; E-mail: taihopark@postech.ac.kr

Contents

Experimental Details

Figure S1. Equivalent circuit models of symmetric cells in the structure of (a) Pt/CCPL/SS and (b) CL/CCPL/SS or CCPL/SS.

Figure S2. Comparison of the adhesion strength of the CL(86 wt%)/CCPL/SS and CL(90 wt%)/CCPL/SS CEs: Pull-off adhesion strength test using a PST. The detached CB are indicated by the yellow dotted circle

Figure S3. Absorption spectra of N719/TiO_2 films used to fabricate devices employing CL/CCPL/SS, Pt/FTO, and Pt/CCPL/SS counter electrodes.

Experimental Details

a. **Preparation of devices.** 20 nm-Sized TiO_2 particles were screen-printed onto the $TiCl_4$-treated FTO surface to produce a transparent active layer (9 μm). The second light-scattering layer (5 μm) was prepared by screen-printing 400 nm-sized anatase particles onto the transparent layer. The thicknesses were measured using an Alpha-Step 500 Surface Profilometer (Tencor Instruments, USA). The double layer prepared on the FTO glass was sintered at 500 °C for 30 min according to a programmed heating procedure. After cooling to 60 °C, the nanocrystalline TiO_2 electrode was immediately immersed in a dye solution to minimize moisture adsorption, kept at room temperature for 18h. For DSCs employing liquid electrolytes, the dye solution of 0.3 mM N719 sensitizer in acetonitrile and tert-butanol (1:1 v/v) was used and sensitized for 18 h at room temperature. The conventional counter electrode (Pt/FTO) was prepared by introducing two holes, using a sandblasting drill, into the FTO conducting glass for the substrate. The Pt paste was spread onto the predrilled FTO conducting glass and subsequently sintered using a programmed heating procedure. The dye coated photoanode and counter electrode were assembled and sealed as a sandwich using a transparent 60 μm thick surlyn spacer (DuPont) by hot pressing. The inter-electrode space was filled with the liquid electrolyte (0.1 M I2, 0.4 M. TBAI, 0.6 M BMImI, 0.1 M GuSCN, and 0.5 M tBP in a mixture solvent of acetonitrile/valeronitrile (85:15 v/v)).

b. **Field emission scanning electron microscopy (FE-SEM) measurement.** Field emission scanning electron microscope (FE-SEM, Hitachi S 4800) was employed for observing the top surfaces and cross section surfaces of the various substrates.

c. **Photovoltaic performance measurement.** A 150 W xenon light source (Model No. 94022A, Oriel) was used to apply an illumination power of 100 $mWcm^{-2}$ (the equivalent of one sun at AM 1.5) to the surface of the solar cell to simulate solar light irradiation. The incident light intensity was calibrated with reference to a Si solar cell equipped with an IR cutoff filter (KG-5, Schott). Comparison of the simulated light to the true solar spectrum in the region 350–750 nm determined a spectral mismatch of less than 2%. The J–V characteristics were obtained by measuring the photocurrent generated by the cells (under an applied external bias) using a Keithley model 2400 digital source meter (Keithley, USA).

d. **Electrochemical impedance spectrophotometer measurement.** The impedance values of devices having different structures were measured using a computer-controlled potentiostat (SP-200, BioLogic) under dark conditions. The frequency range examined was 0.5 Hz–1 MHz at room temperature, and the impedance spectra were recorded 0 V with a voltage amplitude set at 30 mV. The measured spectra were fit to appropriate equivalent circuits using the Z-fit software provided by BioLogic.

e. **Four-point probe measurement.** The resistivity of CCPL on the glass was measured with the four-point probe method (Keithley 2400 source-meter).

e. **Cyclic voltammetry.** Cyclic voltammetry (CV) was carried out in a three electrode one compartment cell with a self-made working electrode, Pt wire counter electrode and an Ag/AgCl reference electrode dipped in an acetonitrile solution of 10 mM LiI, 1 mM I_2, and 0.1 M $LiClO_4$. CV performed using potentiostat (SP-200, BioLogic) electrochemical measurement system (sweep condition: 30 mV/s).

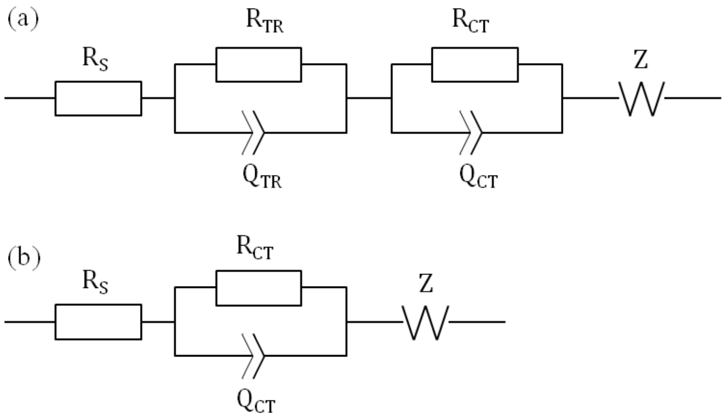

Figure S1. Equivalent circuit models of symmetric cells in the structure of (a) Pt/CCPL/SS and (b) CL/CCPL/SS or CCPL/SS. R_S is the series resistance of the electrode. R_{tr} is electron transfer resistance at the Pt/CB interface and the R_{CT} is charge transfer resistance indicating catalytic activity at the electrolyte/CB or Pt interfaces. Q is the capacitance of the interface regarding a constant phase element (CPE). Z is the Warburg impedance describing the diffusion of I_3^- in the electrolyte.

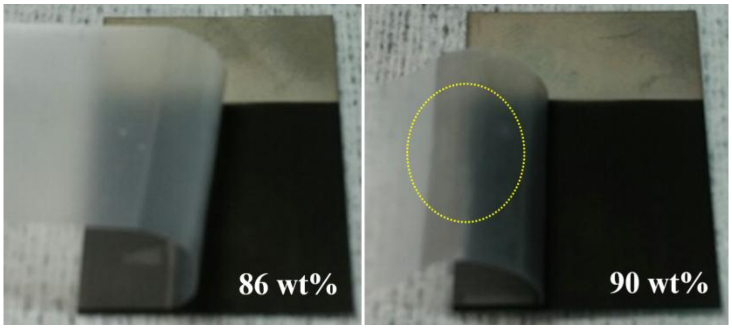

Figure S2. Comparison of the adhesion strength of the CL(86 wt%)/CCPL/SS and CL(90 wt%)/CCPL/SS CEs: Pull-off adhesion strength test using a PST. The detached CB are indicated by the yellow dotted circle

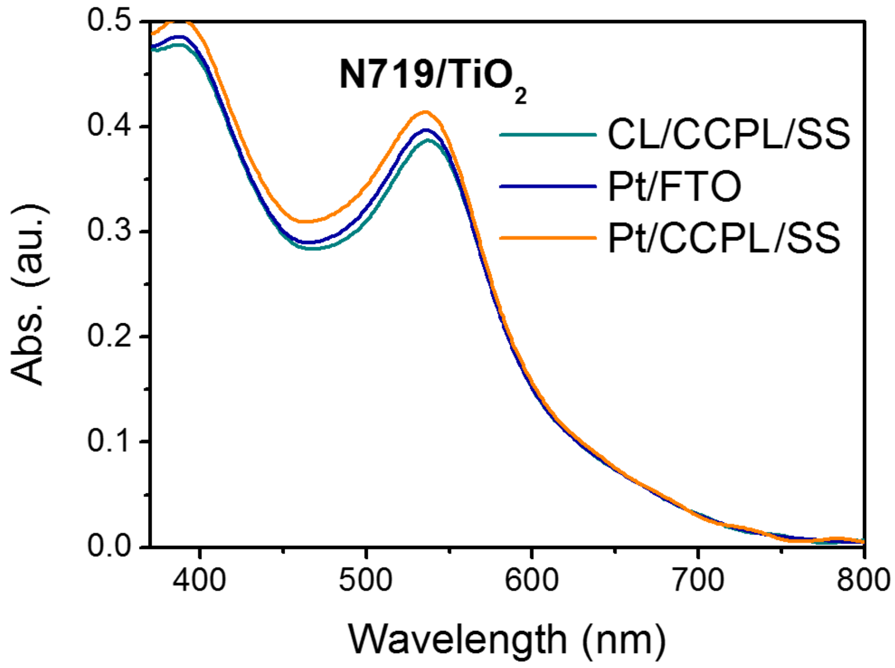

Figure S3. Absorption spectra of N719/TiO_2 films used to fabricate devices employing CL/CCPL/SS, Pt/FTO, and Pt/CCPL/SS counter electrodes.
